# Epilepsy: A Call for Help

**DOI:** 10.3390/brainsci8020022

**Published:** 2018-01-28

**Authors:** Venkatraman Sadanand

**Affiliations:** Department of Neurosurgery, Loma Linda University; Loma Linda, CA 92354, USA

**Keywords:** epilepsy, epilepsy surgery, decision analysis, treatment gap, game theory, economics, efficiency, resource allocation, return on investment

## Abstract

Epilepsy is a considerable individual and social economic burden. In properly selected patients, epilepsy surgery can provide significant relief from disease, including remission. However, the surgical treatment of epilepsy lags in terms of knowledge and technology. The problem arises due to its slow adaptation and dissemination. This article explores this issue of a wide treatment gap and its causes. It develops a framework for a rational decision-making process that is appropriate for extant circumstances and will result in the speedy delivery of surgical care for suitable patients with medically intractable epilepsy.

## 1. Introduction

Epilepsy is known to be a devastating disease not only for the patient but also for the patient’s family and for society. If left untreated, the long-term impact can be significant. If treatment can result in being seizure-free or reduce the severity or frequency of seizures, then it may be worth treating. In fact, surgical treatment is worthwhile, but not everyone who is a good surgical candidate gets timely surgery for their epilepsy. The wait times to see a neurosurgeon are often large. In most countries, the time from diagnosis to referral to an epilepsy center ranges from 18.9 [1] to 20 years [2].

This paper explores why this might be the case. Physicians tend to rightly believe that surgical decision is based solely on medical evidence. This paper argues that this alone is not sufficient. In fact, the eventual decision for surgery of a patient is the end step in a complex decision-making process involving multiple stakeholders. This paper therefore has two tasks: first, to identify the stakeholders and their incentives; second, to model the complex interactions among this group of entities with vested interests. 

For this purpose, the present paper will consider two factors that characterize this analysis. First, it postulates that the primary objective of individual stakeholders is different for each stakeholder and may conflict with those of others. From a purely public health perspective, the objective is to maximize the return on investment (ROI) while maximizing public welfare. Using the ROI approach, we will compare ROI in epilepsy surgery as the use case with ROI in glioblastoma multiforme (GBM) as a control case. The control case could be any disease with postulated lower ROI and higher investments than epilepsy surgery. Second, the complex interactions among stakeholders with diverse objectives working in an environment of strategic interactions is impossible to model using standard decision analysis wherein individual stakeholders are working with fixed outcomes of their actions. In other words, present models portray an individual stakeholder making decisions under the assumption that their decisions directly impact *only* the final outcome. In particular, they do not account for the fact that, in addition, their decisions will also have an impact upon the decisions of other stakeholders who are involved, which will alter the final outcomes. Thus, all stakeholders take actions that affect all other stakeholders, and this inevitably leads to strategic decision-making by each while being cognizant of the interdependence on others, until an equilibrium is reached where everyone obtains an outcome. This is not like previous models where a given fixed net outcome is shared by all the stakeholders (denoted sometimes as a problem of dividing a fixed pie. Those problems are referred to as zero-sum games. The current problem we analyze is not a zero-sum game). In our analysis, when equilibrium is reached, each stakeholder obtains an outcome that is of a specific value to that stakeholder. What we require is a way to model the strategic interactions among multiple stakeholders when each one has an individualized objective that may conflict with those of others in the same environment. Such models of strategic decision-making were first postulated mathematically by John Nash [3] and subsequently developed further through applications to other fields [4]. This field of mathematics is called non-cooperative game theory.

This is the first time non-cooperative game theory is being used in the literature on medical decision analysis. Why is non-cooperative game theory a better tool for medical decision analysis than standard Markov decision trees? The reason is that it is more realistic and hence a more accurate model of stakeholder interaction. The stakeholders are not passive observers in the system as in the current modeling techniques in medical decision analysis. In fact, every stakeholder is a maximizer of his own objective function and, in that process, recognizes and adjusts dynamically to the behavior calculus of every other stakeholder. Each stakeholder will try to predict the behaviors of all other stakeholders in an environment of uncertainty and incomplete information about others. This is the essence of non-cooperative game theory. Economic modeling, market analysis, individual behavior analysis, group dynamics, and military decision-making have all abandoned the previous naïve models of dynamic programming with fixed incentive for players. Instead these models have evolved to game theory. This shift from fixed incentives-based interactions to a dynamic interaction of multiple players with diverse objectives who think through others’ thought processes and adjust on the go is the very basis of a complex mathematical analysis leading to the Nobel Prize in Economics in 1994 [3,4,5]. The mathematics behind such analysis has been referred to as game theory. Even individual biological cell behavior is now seen to follow such models, that have been referred to as biological games. Thus, this paper deviates from existing literature and advocates the use of game theory for medical decision-making and considers the techniques of static and dynamic programming with fixed incentives as a source of misleading results and not a model of real behavior.

Consider the example of a low returns (ROI)–high investment disease such as glioblastoma multiforme (GBM), an aggressively malignant brain tumor. This is also a devastating disease with considerable negative impact on the individual and his or her family. Despite the best treatment options, median survival is about 18 months [6], and most patients who are diagnosed with this disease have low productive horizons ahead of them. 

Current data appears to establish that, if we compare epilepsy with GBM, the incidence of GBM is lower, prevalence is lower, direct treatment costs are higher, indirect treatment costs are higher, benefits are lower, contribution to GDP is lower, and ROI is lower—yet we invest more readily in GBM treatment than in epilepsy surgery. A patient with GBM reaches the surgeon usually within a few days after diagnosis. A patient with epilepsy takes 18–20 years [1,2] from diagnosis to reach the surgeon in most countries. This is a problem.

This chapter addresses this issue, quantifies it, and suggests an approach based on game theoretic analysis to develop a methodology to take a deeper look at the nature of this treatment gap and socio-economic inefficiency. 

In public health, as in public policy, economic efficiency is measured by a concept borrowed from applied mathematics called Pareto optimum [7]. When the welfare of stakeholders can be improved from a given situation without making anyone less well-off than the status quo, it is called a Pareto improvement. It is well established in the economics and public policy literature that economic efficiency requires Pareto optimal outcomes. This well-known result led to the Nobel Prize in 1972 for Kenneth Arrow [8]. More recently, it has been advocated that Pareto optimization cannot be ignored in medical decision-making and public health [9]. Game theory will model realistic interaction among stakeholders, and the theorem of Pareto optimality will yield economic efficiency. Thus, amid paucity, the creation of epilepsy centers of excellence can be Pareto optimal. 

## 2. Problem Definition

The underlying problem explored in this chapter is best described by Table 1, where the two diseases are compared: glioblastoma multiforme (GBM) and surgical epilepsy (SE). SE is 3000 times more prevalent than GBM. There are 400 times more patients who are candidates for SE than for GBM. Total expenditures on research and treatment is roughly the same for both diseases. However, the benefits of surgery per year for a patient with SE is 80,000 times that of a patient with GBM.

In Table 1, the direct costs for epilepsy include neurology specialist consultations, primary care visits for reasons related to the disease, number and type of diagnostic tests performed (basic blood test, analysis of anti-epileptic drug levels, brain Computed Tomography (CT) scan, brain Magnetic Resonance Imagine (MRI), brain Single Photon Emission Computed Tomography (SPECT) and Positron Emission Tomography (PET), Electrocardiogram (ECG), Electroencephalogram (EEG), EEG with sleep deprivation, Holter EEG, Video Electroencephalogram (VEEG), and Holter ECG), days of hospitalization for diagnostic work up, and the treatment administered. Non-medical direct costs include the use of transport to and from hospital and psycho-educational and social support. Costs of anticonvulsant medications were the highest factor in direct costs. Furthermore, the indirect costs of epilepsy are higher than direct costs in most studies. This is because of the value of a productive person in society even if only accounting for a small probability of diminished abilities after surgery. 

In the case of GBM, direct costs include consultations with primary care physicians, neurology or neurosurgery specialists, and diagnostic tests such as CT scans, brain MRI, as well as ECGs and blood tests. Once the diagnosis of a brain tumor is made, the patient goes immediately (during the same admission in most cases) to surgery. Surgery and recovery then become direct costs. The last component of direct costs arise from post-operative care, chemotherapy, radiation therapy, rehabilitation, and a repeat of direct costs for each recurrence that is treated. Indirect costs for GBM are lower than for epilepsy because of the limited life expectancy post-diagnosis. In addition, the average age of diagnosis is greater in GBM compared to epilepsy. Hence the span of productive life lost is also less than for epilepsy.

Waiting times for GBM patients, interestingly, are extremely low compared to epilepsy patients. Median time from diagnosis to surgery is less than 1 week and median times from surgery to adjuvant therapy is 27 days. This is despite the costs reported [13] to be between $50,000 and $92,000 per Quality Adjusted Life Years (QALY), barely making it over the accepted cost-effectiveness thresholds of $50,000 per QALY. 

In the United States of America, 369 resective epilepsy surgeries are being carried out per year on the average, whereas there are about 1 million surgical candidates for epilepsy surgery [21]. In India, with a population approximately 4 times that of the United States, only 734 resective epilepsy surgeries are carried out per year when there are 2.5 million surgical candidates [22]. In China, there are about 1.8 million surgical candidates for epilepsy surgery and only about 2500 epilepsy surgeries are carried out per year [23]. Based on the data from these studies [21,23], China has roughly twice the number of surgical candidates but performs eight times more epilepsy surgeries per year. However, one must be cautious before this finding is applauded. Both countries are still woefully short of treating the number of epilepsy surgery candidates. There is a considerable treatment gap impacting individual and social welfare and that treatment gap is the subject of this paper. It is even more surprising that there is such a treatment gap when the seizure-free rate averaging over all types of seizure surgeries is 68% and about 90% for certain kind of seizure foci [24]. 

We have thus far examined the treatment gap, the cost–benefit ratio, and the time taken from diagnosis to surgical attention as measures of this seeming social inefficiency. We may also consider other measures such as the cost-effectiveness ratio and the return on investment (ROI). 

Any medical intervention that extends lives is measured by the cost-effectiveness ratio. It is the ratio of costs divided by number of years gained. This does not account for the value ascribed to those years. The smaller the number, the more worthwhile the intervention. To calculate this, it should be noted that investment for GBM research and treatment extends life by about 1.5 years [6], while investment for epilepsy extends good quality of life by about 40 years on average. This is a conservative estimate due to the multimodal distribution of incidence (computed from [13] by taking the scaled mean of histograms and subtracting that from the WHO 2015 estimate of life expectancy).

Cost-Effectiveness Ratio (CER) = Direct and Indirect Costs in US$/Benefits in Years Gained

For GBM, the CER: 1435 × 10^8^/1.5 = 956 × 10^8^. 

For Epilepsy, the CER is: 1700 × 10^8^/40 = 42.5 × 10^8^. 

The Benefit Cost Ratio (BCR) is like the CER but considers the value of life lived. This then becomes a proxy for the ROI and has been computed and is shown in the last column of Table 1. 

While there are several other measures one could use, such as the QALY, the point is clear: SE research and treatment, compared to GBM research and treatment, is more efficient, more efficacious, better for the patient, and better for the society. However, SE treatment is gravely underutilized. Thus, how is this market inefficiency explained and how can this be addressed? A purely medical decision-making is an evidence-based decision. On that basis alone, there is no paucity of evidence to show that, for the properly identified candidate, epilepsy surgery is the right option rather than medical management. However, this would ignore the reality of a medical economy. One must review the incentives and disincentives of all stakeholders in this game of budget allocation for surgery to advance the patient from diagnosis to surgery. Just because medical evidence shows that epilepsy surgery is better for properly selected patients compared to medical management, it does not mean that those patients will receive surgery in an environment where there are several stakeholders with conflicting and diverse goals.

## 3. The Stakeholders, Their Incentives and Pitfalls

Surgical decision-making in epilepsy involves several stakeholders along the path from diagnosis to surgery. The first is the physician. The second is the patient and his or her family. The third is hospital administration. The fourth is the insurance industry. The fifth is society. 

The physician’s decisions are typically evidence-based. This is a necessary condition for surgery but not sufficient. However, their role can push to decrease the large gap of almost 20 years from diagnosis to surgery and, once cleared for surgery, to push for appropriate budget allocations to enable surgery. The doctor, in most epilepsy centers, must take on the role of a physician and that of a patient advocate. 

Next, the patient and his family are dealing with a disease that not only results in a loss of income and productivity but also the social stigma and discrimination associated with epilepsy. Therefore, they need to be educated in the social and quality of life advantages of epilepsy surgery. Such patient education is glaringly lacking until the patient somehow makes his first contact with a doctor. In some countries, epileptologists are usually based in urban centers while many epilepsy patients are in rural areas.

Hospital administrations must balance budgets and face multiple demands from different subspecialties. Resource allocation then becomes an issue of educating administrators about the cost-effectiveness and efficacies inherent in epilepsy surgery, even when compared to other specialties that may have their own demands for fixed budgets, finding a proper fit with corporate missions and medicolegal experiences.

Insurance industry needs to be made aware of the cost–benefit analysis of surgery versus the medical care of epilepsy patients. In computing these costs and benefits, care must be taken to account for the discounted present values along with the non-zero rates of return. Insurance companies therefore must now weigh the cost of surgical care against the discounted present value of the future cost of medical care. Social welfare and lost productivity are often not in their optimization problem and may be considered externalities [25]. Externalities impose costs on other stakeholders but have no impact on the given stakeholder’s mathematical profit maximization function.

The last stakeholder is society. How does one account for the value of epilepsy surgery to society? Epilepsy surgery creates productive people who can live healthy lives. That is the very basis of measures such as BCR and ROI. Thus, the impact of epilepsy surgery on this last stakeholder is measured by the value of an individual on society. The current belief is that the value of an individual’s life is measured by the market value of the goods and services produced by that individual. However, this may be incorrect. The value of an individual’s productivity to society may exceed the actual productive output of the individual due to social multiplier effects and what is known as Okun’s law [26,27], and this must also be accounted for in computing society’s objectives in this interactive strategic decision analysis. 

## 4. Decision Analysis

What we have now is a complex problem of resource allocation with the following characteristics: the existence of multiple strategic stakeholders (so current models of a single stakeholder such as a physician, making medical evidence-based decisions alone is an inadequate model), diverse goals (so a multiple stakeholder decision analysis wherein all stakeholders have the same goal of patient welfare is also inadequate), computational complexities of payoffs to each stakeholder (so a model of multiple stakeholders with fixed objectives is inadequate), the interdependence of multiple stakeholders wherein one stakeholder’s decisions affects not only his own but also the outcomes of others (hence a model where a stakeholder’s choices only affect his outcome is inadequate) and the freedom to choose what is best for each stakeholder (so a model of decision analysis that ignores the strategic freedom to choose for each stakeholder is also inadequate). In this environment, it would not be helpful to model this situation as a simple static evidence-based decision for the surgeon. Firstly, the surgeon is not the only stakeholder. Secondly, not every stakeholder has the same objective and incentives as the surgeon. Lastly, not every stakeholder fully knows what others know. This is the reason why models of what ought to be the outcome (surgery, in this case) often differs from what becomes the outcome (treatment gap, in this case). 

The process of medical decision-making is therefore best described and computed by what is known as strategic game theory [28]. Each stakeholder is working in an environment of incomplete information (not everyone knows what others fully know) about the incentives faced and considered by other stakeholders and uncertainty about their actual decisions. A decision analysis will create a game of imperfect information in which all the stakeholders choose strategies to maximize their gains under uncertainty in the environment and under uncertainty about others’ choices. Such complex interactions may involve coalition forming among the stakeholders and may even result in sharing gains from the outcome.

In such games, every player first defines what his or her goal is and what would be the mathematical representation of those goals. It then seeks to maximize that mathematical entity while accounting for other stakeholders’ similar thought processes. Outcomes in such situations are called equilibria of games [29]. Since every player functions under some degree of uncertainty about the other players’ motives and moves, the final action of each stakeholder is dictated by what is known as Bayesian expected utility and interactive equilibrium [29]. This is the decision-making process used by most Forbes 100 businesses, and it may be about time that medical decision-making follows this approach by considering the strategic nature, the informational asymmetries, uncertainties in the environment and the diverse goals of stakeholders.

## 5. Conclusions

There is medical evidence that, for carefully selected candidates, surgery is better than medical management for patients with medically intractable epilepsy. However, SE is underutilized and there is a treatment gap. SE patients must wait a long time after diagnosis to be referred to a neurosurgeon, resulting in patients who do not receive the surgeries they need. This is not the case for GBM. Patients with diagnosed GBM have waiting times for referral for surgery of a few days, whereas epilepsy patients can wait years. In addition, almost every patient with a first-time diagnosis of possible GBM undergoes surgery. 

The problem may stem from complex interactions of several stakeholders. This paper advocates a method of analysis, called mathematical game theory, that has been used in the fields of business, economics, public policy, political science, and engineering and biology. 

Decision-making for GBM surgery is much easier as all stakeholders appear to focus on the short life expectancy after the diagnosis of GBM and the apprehensive determination to do something to change that. 

It would seem, that fear of death drives decisions differently than hope of improving the quality of long-term life. 

## Figures and Tables

**Table 1:** Cost-Benefit Comparison of GBM versus SE.

Disease	Prevalence (Global)	Incidence (Global)	Surgical Candidates (Global)	Median Survival	Age Distribution of Disease	Costs: Direct/Year in US$ (Research, Prevention, Treatment)	Costs: Indirect/Year in US$ (Individual, Family, Society)	Benefits: Per Year in US$ (Estimated Value of Productive Life)	Benefit/Cost Ratio (ROI Equivalent)
Glioblastoma Multiforme (GBM)	1.6 × 10^4^ [10]	5.0 × 10^4^ [11]	5.0 × 10^4^ [11]	1.5 years [6]	45–70 [6]	1045 × 10^8^ [12,13]	390 × 10^8^ [14]	20 × 10^8^ [15]	0.01
Surgical Epilepsy (SE)	50 × 10^6^ [16]	2.4 × 10^6^ [16]	20 × 10^6^ [16]	60 years [17]	Multimodal [18]	250 × 10^8^ [19]	700 × 10^8^ [19,20]	160 × 10^12^ [15]	1684
